# Relationship of smoking cessation duration and cognitive function among middle-aged and older adults in China: a national cross-sectional study

**DOI:** 10.3389/fpubh.2024.1503152

**Published:** 2025-01-07

**Authors:** Wenhang Zuo, Jin Peng, Jinhui Wu

**Affiliations:** Center of Gerontology and Geriatrics, National Clinical Research Center for Geriatrics, West China Hospital, Sichuan University, Chengdu, Sichuan, China

**Keywords:** population aging, cognitive function, smoking cessation, duration, China

## Abstract

**Background:**

Population aging and smoking are both major challenges worldwide, particularly in developing countries. We preliminarily explored the relationship of smoking cessation duration and cognitive function among middle-aged and older Chinese.

**Methods:**

The data comes from China Health and Retirement Longitudinal Study (CHARLS) wave 4. Smoking information was collected by standardized questionnaire. Global and memory-specific cognitive functions were assessed. We used restricted cubic spline to explore curvilinear relationship. After discretizing the duration of ex-smokers (quitting ≤2 years, 3–8 years, 9–19 years, and ≥20 years), multiple linear regression models were established with current smokers as reference.

**Results:**

A total of 5,561 respondents (67.7 ± 6.2 years; 54.1% men) were included. Respondents who quit smoking for longer showed better global cognitive function. This trend leveled off among respondents who had quit smoking for ≥20 years. There were significant differences in global cognitive function among those who quit smoking for ≥9 years (9–19 years, *β* = 0.75, 95%CI 0.32–1.18; ≥20 years, *β* = 0.94, 95%CI 0.42–1.46). The differences mainly came from men. In immediate memory, all ex-smokers performed better. In delayed memory, only those quit for ≥9 years had significant differences.

**Conclusion:**

Compared with current smokers, both never smokers and ex-smokers showed better cognitive function. Respondents who quit smoking for longer had better cognitive performance, especially those who had quit for at least 9 years.

## 1 Introduction

Population aging is a prevalent global phenomenon. It is estimated that 1/6 people in the world will be over the age of 65 by 2050 (1). China is a country with the largest aging population and the fastest aging speed in the world. According to data from the Seventh National Population Census of China in 2020, there were 264 million people aged 60 and above in China, of whom 191 million were over 65 years old (2). With severe population aging, the prevalence of cognitive impairment continues to increase. Cognitive function includes domains such as attention, executive function, memory and so on. Cognitive impairment occurs when one or more domains are impaired. According to data from 2015 to 2018, the prevalence of mild cognitive impairment among Chinese older adults was about 15.5%, and the prevalence of all-cause dementia was about 6.0% of them (3). Cognitive impairment reduces the quality of life and shortens the life expectancy of older adults.

Tobacco is currently one of the most significant health threats in the world. In 2020, the World Health Organization estimated that 22.3% of global population (36.7% men and 7.8% women) were using tobacco, and more than 80% of them live in low-income and middle-income countries, with the heaviest burden of tobacco-related diseases and deaths (4). China is the largest tobacco producer and consumer in the world. The *2018 Chinese Adult Tobacco Survey* showed that the number of current smokers exceeded 300 million, with smoking rates of 30.2 and 23.1% among middle-aged and older adults, respectively (5). Smoking is not only closely associated with chronic diseases but also closely related to geriatric syndromes such as sarcopenia, disability, and frailty (6–9). After a long period of controversy, it is now generally accepted that long-term smoking is harmful to cognitive function (10, 11).

At present, we still lack effective means to reverse chronic cognitive impairment. As a modifiable behavior, quitting smoking may be a way to reduce the risk of cognitive impairment. Some studies, all from developed countries, evaluated cognitive function after smoking cessation (12–16). Given the more severe tobacco-related burden and the lack of social and medical resources in developing countries, the risk of cognitive impairment in these populations should receive more attention. This is particularly significant for countries like China, which are concurrently confronting a notable aging demographic for whom the relationship between smoking cessation duration and cognitive function remains unclear. Therefore, we conducted a cross-sectional analysis using a nationally representative survey of middle-aged and older residents in Chinese communities to explore the relationship between smoking cessation duration and cognitive function.

## 2 Materials and methods

### 2.1 Data and study sample

We used cross-sectional data from the fourth wave of the China Health and Retirement Longitudinal Study (CHARLS) conducted in 2018. The program initiated a national baseline survey in 2011 and conducted follow-up surveys every 2 or 3 years. At baseline, a stratified multistage probability proportionate to size sampling (PPS) strategy was used to randomly select community residents aged 45 or over from 28 provinces in China (17, 18). The CHARLS program was approved by the Institutional Review Board of Peking University (No. IRB00001052-11015) and the written informed consent was obtained from all respondents. This study was exempt from the approval of the institutional review board, because all original data was de-identified and publicly accessed through the CHARLS website.^1^ This study was performed in line with the principles of the Helsinki Declaration.

**Figure 1:**
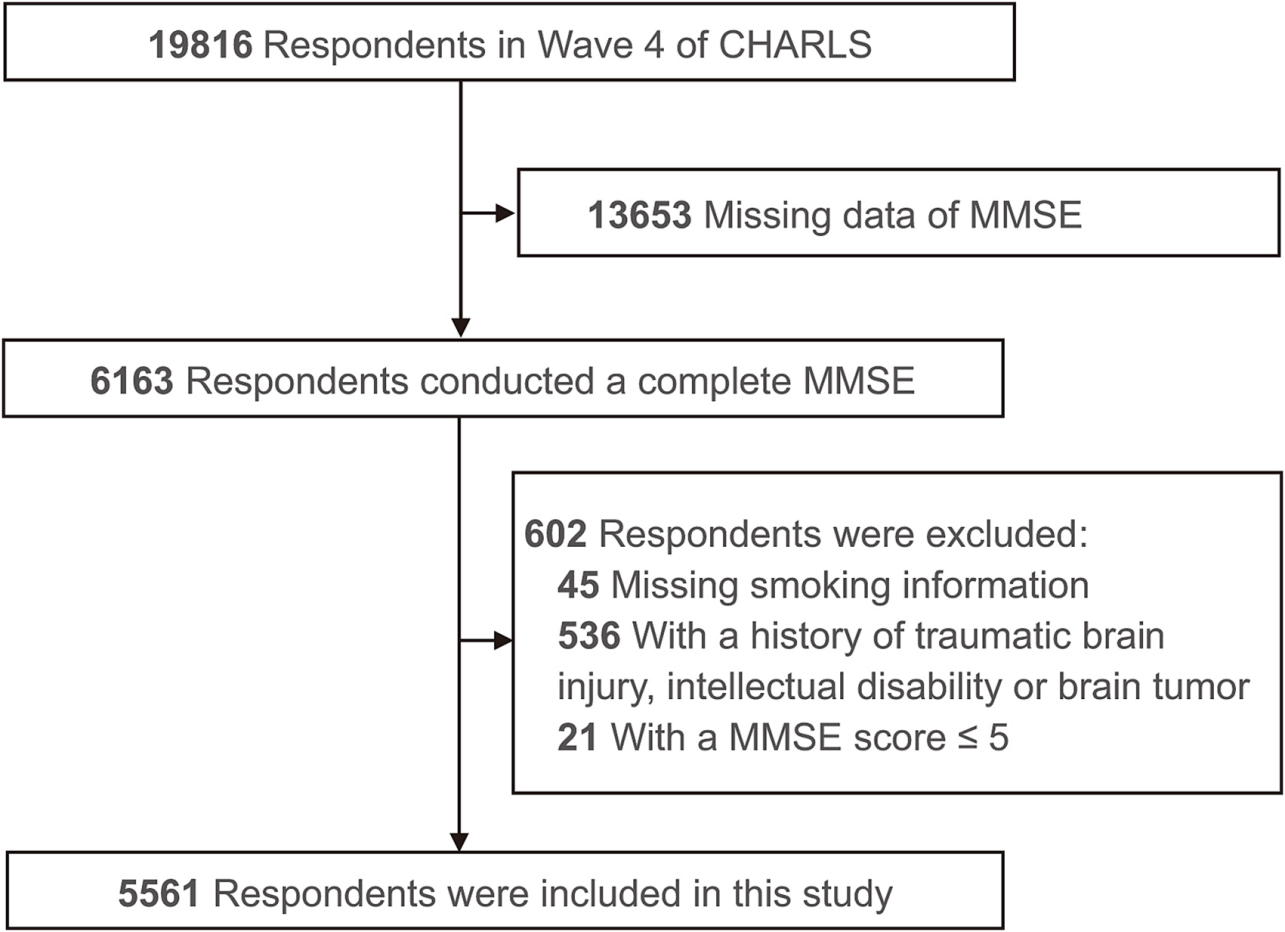
Flowchart of study sample. CHARLS, China Health and Retirement Longitudinal Study; MMSE, Mini Mental State Exam.

In the fourth wave of CHARLS, a total of 6,163 respondents had a complete score for global cognitive function. Those lacking smoking information [such as smoking history, current smoking status, and smoking cessation duration (*n* = 45)] were excluded, as were 536 respondents with at least one history of traumatic brain injury, intellectual disability or brain tumor, and 21 respondents whose cognitive function was so impaired as to hinder the accuracy of their questionnaire and assessment. The final study sample included 5,561 respondents (Figure 1).

### 2.2 Smoking information

In each wave of CHARLS, trained and qualified investigators used standard questionnaires to collect respondents’ self-reported smoking information. Briefly, it included the following questions. (1) Have you ever smoked? By smoking we mean smoking more than 100 cigarettes in your lifetime. (2) Do you still have the habit or have you totally quit? (3) Age or year when smoking started. (4) Which products do/did you normally use? (5) How many cigarettes do/did you smoke on average a day? (6) At what age or year did you totally quit smoking? In this study, respondents who smoked filtered cigarettes accounted for 97% of all smokers. We used retrospective smoking information obtained at all surveys to reconstruct a more complete lifetime smoking history at Wave 4.

The smoking status at Wave 4 included three categories: never smokers, current smokers and ex-smokers. According to the results of restricted cubic spline (RCS) in this study, we further divided ex-smokers into four groups: quitting for ≤2 years, 3–8 years, 9–19 years, and ≥20 years. Consistent with previous studies, pack-years were used to represent cumulative smoking amount (13, 14, 19). Pack-years were equal to years of smoking multiplied by the average number of packs smoked per day during these years, assuming that each pack contained 20 cigarettes.

### 2.3 Cognitive function

In our study, Mini Mental State Exam (MMSE) was used to evaluate global cognitive function. The Chinese version of MMSE has good reliability and validity in CHARLS cohort (17, 20). Trained and qualified investigators conducted a face-to-face cognitive assessment of respondents. It totals 30 points, and a higher score means better cognitive function. Scores ≤5 points indicate severe cognitive impairment.

Memory is one of the core domains of cognitive function. As secondary indicators in our study, immediate and delayed word memory tests from the Consortium to Establish a Registry for Alzheimer’s Disease (CERAD) were used to demonstrate the function of episodic memory. It totals 20 points, including immediate word memory (10 points) and delayed word memory (10 points). A higher score indicates better memory.

### 2.4 Covariates

The CHARLS team utilized a standardized and procedural questionnaire to collect socio-demographic information, lifestyle habits, and health status. Combining clinical experience and literature, we included possible relevant factors into stepwise regression (with inclusion and exclusion thresholds set at 0.2) to identify significant variables. These variables included age (years), sex assigned at birth (male or female, obtained from the household registration form), education (illiterate, primary school, middle school, or ≥high school), marital status (married/cohabiting, or other statuses), residence (urban or rural), current alcohol intake, depressive symptoms, number of chronic diseases, chronic pain, socially active, and cumulative smoking amount.

Respondents were considered as having current alcohol intake if they reported consuming alcohol on average more than once a month in the past year. The Center for Epidemiological Studies Depression Scale short form (CES-D10) was used to evaluate depressive symptoms in the CHARLS. Depressive symptoms were defined as having a CES-D10 score of 12 or above (21, 22). Chronic diseases included self-reported and physician-diagnosed hypertension, diabetes, dyslipidemia, cancer or malignant tumor (excluding minor skin cancer), heart disease, chronic lung disease, chronic kidney disease, chronic liver disease (excluding fatty liver), chronic digestive disease, arthritis, and rheumatism. Respondents who were undergoing medication treatment for hypertension, diabetes, or dyslipidemia were also considered as having diseases. Respondents were asked “Do you often feel pain in any part of your body?” Those who answered “a little,” “quite a lot” or “very much” were considered to have chronic pain. Socially active was considered as having participated in at least one social activity within the month prior to the survey.

### 2.5 Statistical analysis

Descriptive analysis was employed to illustrate the characteristics of respondents, as well as to examine differences between groups. Continuous variables with a roughly normal distribution were reported as mean and standard deviation. Intergroup differences were assessed using analysis of variance (ANOVA), followed by Bonferroni’s multiple comparison test for pairwise comparisons among groups. Skewed data (such as cumulative smoking amount) were presented using median and interquartile range, and intergroup comparisons were performed using Wilcoxon rank-sum test. Categorical variables were reported as frequency and percentage, and intergroup differences were evaluated using Pearson’s chi-squared test. The significance level was set at *α* = 0.05.

To visually demonstrate the non-linear association between smoking cessation duration and global cognitive function, we employed an RCS analysis. We set four nodes at 25, 50, 75, and 90% of the years since smoking cessation, which corresponded to 2, 7, 16, and 26 years, respectively. Using global cognitive function of current smokers (years from smoking cessation was 0) as a reference and adjusting for all covariates, we generated a restricted cubic spline plot.

Referring to the graphical trends of RCS, we chose 9 and 20 years of smoking cessation as grouping nodes. Additionally, to investigate the short-term effects of smoking cessation, we also included 2 years as a grouping node. The ex-smokers were then stratified into four groups based on the duration of smoking cessation: ≤2 years, 3–8 years, 9–19 years, and ≥20 years. A multiple linear regression model was established, with the cognitive score as dependent variable, smoking cessation duration and covariates as independent variables. The regression coefficients (*β*), 95% confidence intervals (CI), and *p*-values were recorded for each group compared to current smokers (years from smoking cessation was 0).

To explore the robustness and heterogeneity sources of main results, we adjusted for various covariates in models, as well as conducted subgroup analyses and complete data analyses. In the complete data analysis, respondents with missing values in any covariate were excluded, and a model adjusting for all covariates was established using complete data. Stata/SE 16.0 was used to conduct the statistical analysis.

## 3 Results

### 3.1 Characteristics of the respondents

A total of 5,561 respondents were included in this study, and they were categorized into three groups based on smoking status: never-smokers (*n* = 2,802), current smokers (*n* = 1,608), and ex-smokers (*n* = 1,151). The characteristics of each group and the results of intergroup comparisons are presented in Table 1. Never-smokers were more likely to be female and had lower levels of education, marriage rates, and alcohol consumption. Furthermore, they were more likely to suffer from depression symptoms and chronic pain. Males constituted the majority of both current smokers and ex-smokers. Compared to current smokers, ex-smokers were older on average, more likely to reside in urban areas, had more kinds of chronic diseases, as well as had lower rates of alcohol consumption and cumulative smoking exposure.

**Table 1:** Characteristics of study populations by smoking status.

	Overall (*n* = 5,561)	Never smoker (*n* = 2,802)	Current smoker (*n* = 1,608)	Ex-smoker (*n* = 1,151)	^*^P-value	^†^*P-*value
Age, mean (SD), y	67.7 (6.2)	67.6 (6.2)	66.8 (5.7)	69.0 (6.7)	<0.001	<0.001
Male, *n* (%)	3,010 (54.1)	514 (18.3)	1,458 (90.7)	1,038 (90.2)	<0.001	0.666
Education, *n* (%)	—	—	—	—	<0.001	0.292
Illiterate	1,035 (18.6)	752 (26.8)	162 (10.1)	121 (10.5)	—	—
Elementary school	2,610 (46.9)	1,239 (44.2)	802 (49.9)	569 (49.4)	—	—
Middle school	1,162 (20.9)	474 (16.9)	416 (25.9)	272 (23.6)	—	—
≥High school	754 (13.6)	337 (12.0)	228 (14.2)	189 (16.4)	—	—
Married/living together, *n* (%)	4,607 (82.8)	2,212 (78.9)	1,397 (86.9)	998 (86.7)	<0.001	0.896
Residence in urban, *n* (%)	2,504 (45.0)	1,330 (47.5)	645 (40.1)	529 (46.0)	<0.001	0.002
Current alcohol intake, *n* (%)	1,573 (28.3)	357 (12.7)	784 (48.8)	432 (37.5)	<0.001	<0.001
Smoking amount, M(IQR)^‡^	32.3 (32.5)	—	35.0 (29.5)	29.5 (37.0)	—	0.001
Socially active, *n* (%)	2,567 (46.2)	1,310 (46.7)	744 (46.3)	513 (44.6)	0.455	0.377
Depressive symptoms, *n* (%)	1,470 (26.4)	854 (30.5)	377 (23.4)	239 (20.8)	<0.001	0.095
Chronic pain, *n* (%)	1,540 (27.7)	900 (32.1)	363 (22.6)	277 (24.1)	<0.001	0.360
Chronic diseases, mean (SD)	2.3 (1.7)	2.4 (1.8)	2.0 (1.6)	2.5 (1.7)	<0.001	<0.001
MMSE score, mean (SD)	22.5 (5.1)	21.7 (5.5)	23.2 (4.5)	23.6 (4.6)	<0.001	0.101
Episodic memory, mean (SD)^§^	7.0 (4.1)	6.9 (4.3)	6.8 (3.8)	7.3 (3.9)	0.014	0.015

*P-value, results among the three groups. ^†^*p-*value, results between current smokers and ex-smokers. ^‡^Missing *n* = 84. The sample sizes for each group were 2,802, 1,576, and 1,099, respectively. ^§^Missing *n* = 434. The sample sizes for each group were 2,558, 1,494, and 1,075, respectively. M(IQR), media (inter-quartile range).

### 3.2 Non-linear relationship between smoking cessation duration and global cognitive function

**Figure 2:**
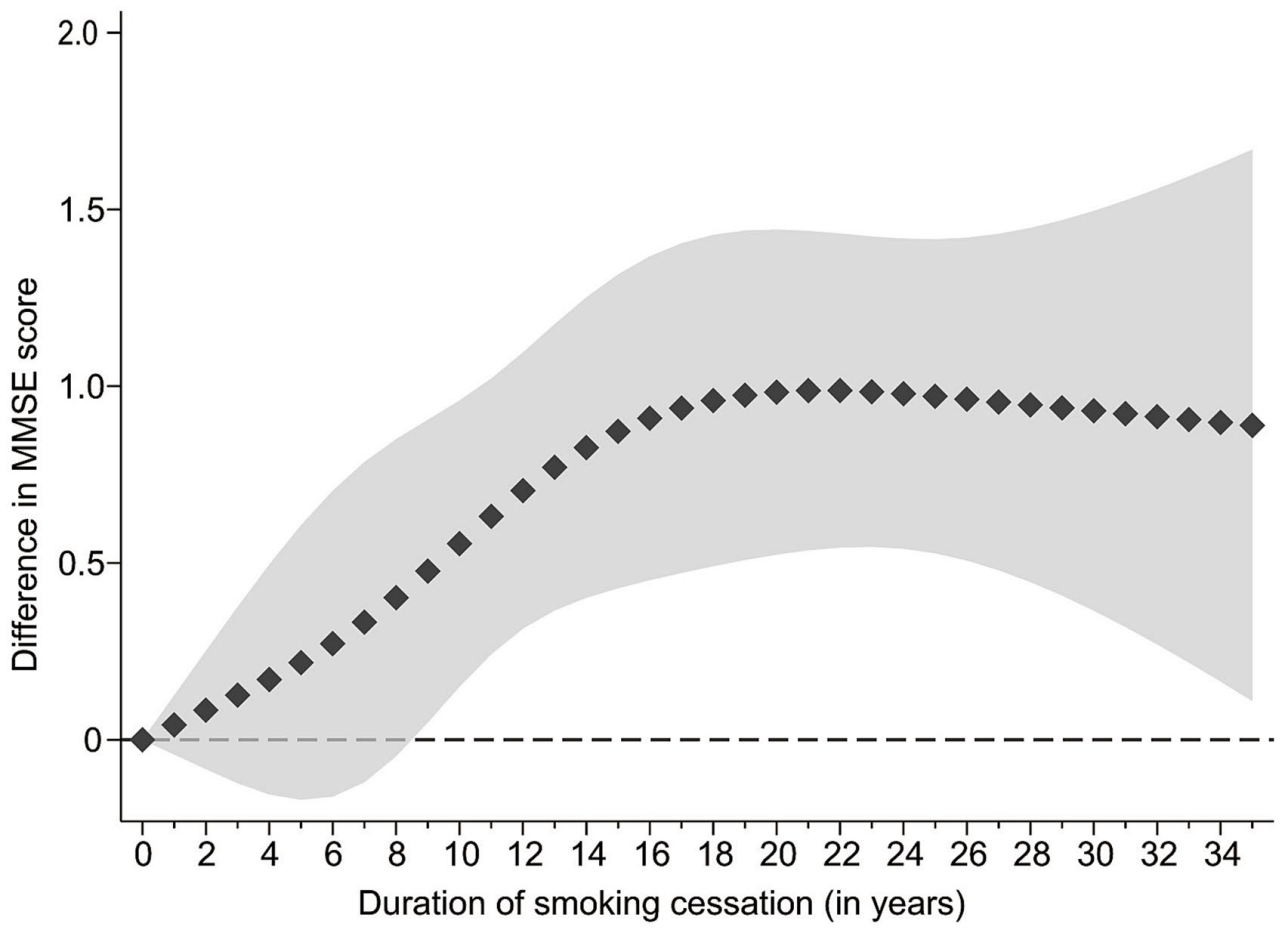
Association between smoking cessation duration and MMSE score in ex-smokers versus current smokers, results of restricted cubic splines analyses. The line composed of squares is the value of difference, and the shaded area is 95%CI.

Figure 2 demonstrated a significant non-linear relationship between smoking cessation duration and global cognitive function. As the trend suggested, respondents who had quit smoking for a longer period of time had better global cognitive function within approximately 20 years of smoking cessation. However, among respondents who had quit smoking for over 20 years, their cognitive function did not differ significantly, and the curve tended to flatten. Although the curve showed an upward tendency within 9 years of smoking cessation, the difference in cognitive function scores between ex-smokers and current smokers was not statistically significant. Only respondents who had quit smoking for 9 years or more exhibited a significant difference in cognitive function compared to current smokers.

Accordingly, we selected 9 and 20 years of smoking cessation as grouping nodes to categorize ex-smokers. The characteristics of each group and the results of intergroup comparisons are presented in Supplementary Table S1. The group with longer smoking cessation duration was likely to have a greater age and reside in urban areas.

### 3.3 Smoking status and global cognitive function

**Figure 3:**
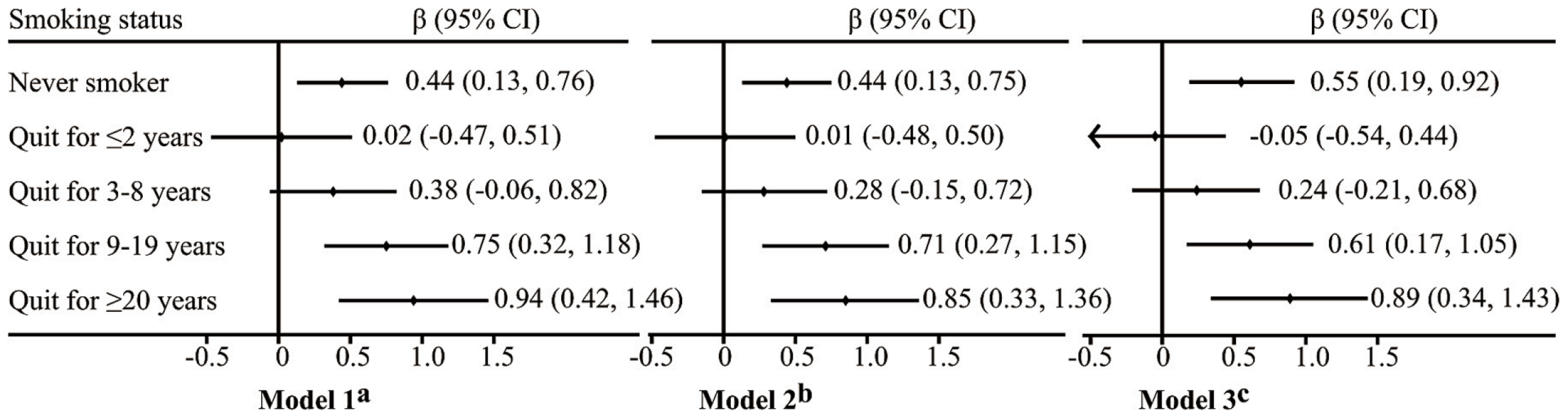
Difference in MMSE score with current smokers as reference, results of multiple linear regression models. ^a^Adjusted for age, sex, education, marital status and residence. ^b^Adjusted for all covariates in Model 1, alcohol intake, socially active, depressive symptoms, chronic pain and chronic diseases. ^c^Adjusted for all covariates in Model 2 and cumulative smoking amount in pack-years (missing *n* = 84).

Figure 3 presents the relative cognitive performance of respondents with different smoking statuses, compared to current smokers as the reference group. In Model 1, we adjusted only for demographic characteristics (i.e., age, sex, education level, marital status, and residence), and the results indicated the following. (1) Compared to current smokers, never smokers had better global cognitive function (*β* = 0.44, 95% CI 0.13–0.76); (2) In comparison to current smokers, ex-smokers generally displayed superior cognitive performance, although the difference for those who quit smoking for ≤8 years was not significant (*p* ≥ 0.05). The global cognitive function of short-term quitters (≤2 years) was almost identical to that of current smokers (*β* = 0.02, 95% CI −0.47 to 0.51). Nonetheless, the difference became significant in the group who had abstained from smoking for at least 9 years (*p* < 0.002). The β (95% CI) was 0.75 (0.32–1.18) for the group who had quit smoking for 9–19 years, and 0.94 (0.42–1.46) for the group who had quit smoking for ≥20 years. (3) Consistent with the trend indicated by the restricted cubic splines, groups who had abstained from smoking for increasingly prolonged periods demonstrated better cognitive performance.

We added lifestyle and health related variables (i.e., current alcohol intake, socially active, depressive symptoms, chronic pain, and number of chronic diseases) in Model 2. The *β* values for each group decreased slightly, but the results remained consistent with the trend and significance of Model 1. Since smoking dosage may be an important confounding factor in the relationship of smoking cessation duration and cognitive function, we added cumulative smoking amount (pack-years) in Model 3. The results showed that even after adjusting for cumulative smoking amount, the trend and significance of their relationship remained relatively consistent.

In the respondents with a history of smoking, the majority were male (90.5%). And it is generally believed that the impact of smoking on cognitive function is a chronic process. Therefore, we selected the following three subgroups to construct multiple linear regression models. As shown in Table 2, the results were basically similar to the above. In the first subgroup analysis, only male respondents (*n* = 2,946) were included. After adjusting for all covariates, the results indicated that there might be a greater difference in cognitive function of never smokers and ex-smokers quitting for ≥20 years compared to current smokers. The second subgroup analysis excluded people who had smoked for less than 10 years (*n* = 135), and the results were basically consistent with the previous results. The third subgroup analysis, which focused on a sample of male respondents who were either non-smokers or had a smoking history of ≥10 years (*n* = 2,908), yielded results that were consistent with the findings of the first subgroup analysis. When we only included females in the model, it showed that there was no significant difference in cognitive function between current smokers and the other groups. Generally speaking, males and respondents who smoked for a long time made major contributions to the results.

**Table 2:** Subgroup and complete data analysis: difference in MMSE score with current smokers as reference, results of multiple linear regression models.

Smoking status	*β* (95% CI)			
	Subgroup 1^†^	Subgroup 2^‡^	Subgroup 3^§^	Complete data^||^
Current smoker	Ref.	Ref.	Ref.	Ref.
Never smoker	0.64 (0.24, 1.04)^*^	0.47 (0.11, 0.83)^*^	0.63 (0.23, 1.04)^*^	0.59 (0.23, 0.96)^*^
Quit for ≤2 years	−0.15 (−0.67, 0.36)	−0.03 (−0.52, 0.46)	−0.15 (−0.67, 0.37)	0.03 (−0.46, 0.51)
Quit for 3–8 years	0.35 (−0.11, 0.81)	0.25 (−0.20, 0.70)	0.36 (−0.11, 0.82)	0.25 (−0.20, 0.69)
Quit for 9–19 years	0.64 (0.11, 1.17)^*^	0.58 (0.06, 1.09)^*^	0.64 (0.11, 1.17)^*^	0.56 (0.12, 1.00)^*^
Quit for ≥20 years	1.00 (0.55, 1.46)^*^	0.92 (0.44, 1.39)^*^	1.04 (0.56, 1.52)^*^	0.83 (0.29, 1.38)^*^

*p < 0.05. ^†^Including males (*n* = 2,946, all covariates were adjusted except for sex). ^‡^Including never smokers and smokers with a history of smoking up to 10 years (*n* = 5,426, all covariates were adjusted). ^§^Including males who have smoked for up to 10 years (*n* = 2,908, all covariates were adjusted except for sex). ^||^Including respondents with complete data (*n* = 5,313, all covariates were adjusted).

In complete data analysis, we excluded respondents with missing values for covariates, and included a total of 5,313 samples. A multiple linear regression model was established and all covariates were adjusted. The results, shown in Table 2, were essentially identical with the findings from primary analysis.

### 3.4 Smoking status and episodic memory

Table 3 presents the episodic memory of respondents in different smoking states, with reference to current smokers. Compared with current smokers, never smokers had better performance in immediate memory (*β* = 0.20, 95% CI 0.04, 0.37), but not in delayed memory. Compared to current smokers, ex-smokers showed a similar trend in episodic memory as global cognitive function. Respondents with longer smoking cessation duration exhibited better episodic memory. Among ex-smokers who quit smoking for less than 8 years, the difference was significant only in immediate memory, but not in delayed memory. Among ex-smokers who quit smoking for ≥9 years, the difference was significant both in immediate and delayed memory.

**Table 3:** Difference in episodic memory with current smokers as reference, results of multiple linear regression models.

Smoking status	*β* (95% CI)		
	Episodic memory	Immediate memory	Delayed memory
Current smoker	Ref.	Ref.	Ref.
Never smoker	0.33 (−0.01, 0.74)	0.20 (0.04, 0.37)^*^	0.12 (−0.11, 0.35)
Quit for ≤2 years	0.37 (−0.07, 0.81)	0.22 (0.01, 0.43)^*^	0.15 (−0.15, 0.45)
Quit for 3–8 years	0.38 (−0.06, 0.80)	0.23 (0.03, 0.44)^*^	0.15 (−0.14, 0.44)
Quit for 9–19 years	0.60 (0.14, 1.06)^*^	0.27 (0.05, 0.50)^*^	0.33 (0.02, 0.63)^*^
Quit for ≥20 years	0.83 (0.28, 1.38)^*^	0.33 (0.08, 0.59)^*^	0.50 (0.12, 0.87)^*^

*p < 0.05. Adjusted for all covariates (*n* = 5,047).

## 4 Discussion

We utilized data from a nationwide survey to conduct a cross-sectional analysis. It was found that never smokers and ex-smokers performed better cognitively than current smokers. Additionally, respondents who had quit smoking for longer durations had better cognitive performance, especially those who had quit for 9 years or more. This association was mainly observed in males and those who had smoked for a long time. As far as we know, this is the first study to assess the relationship of smoking cessation duration and cognitive function among the Chinese population.

Although our findings support the prevailing view that smokers had worse cognitive function, there had been ongoing controversy regarding this matter in the past. Early studies shown that smoking could reduce the risk of developing dementia (10). Then, most of subsequent cohort studies independent of tobacco industry showed that smoking was a risk factor for cognitive impairment (10, 11, 23, 24). North et al. conducted a meta-analysis of observational studies, including nine cohorts with a total of 26,692 individuals of European descent. It revealed that smoking was generally associated with poor cognitive function (11). In a study of the Chinese population, Song Ge et al. included 16,892 participants and found that current smokers had poorer cognitive function compared to never smokers (24).

With further research on the pathological mechanisms, we can gain a clearer understanding of whether smoking cessation can provide cognitive protection at a pathological level. Nicotine is a crucial constituent of tobacco and serves as an agonist for nicotinic acetylcholine receptor (nAChR). After smoking, nicotine rapidly crosses the blood–brain barrier and exerts acute enhancement on cognitive function (25, 26). Although nicotine might induce nAChR expression (27), studies in mice found that nicotine did not prevent or reverse the age-related reduction in nAChR (28). And long-term exposure to nicotine can lead to functional impairment of nAChR in the brain (29–31). Nicotine can also inhibit mitochondrial energy metabolism and alter the glycolytic pathway (32). Besides, Tobacco smoke contains high levels of free radicals and other reactive oxidants, which contributes to neurodegeneration and deposition of *β*-amyloid protein (33). Other components in tobacco smoke (such as transition metals, carbon monoxide, aldehydes, N-nitrosamines, etc.) can result in endothelial dysfunction and vascular damage (34, 35), which are key steps in the development of cognitive impairments caused by vascular pathology (36).

Therefore, smoking cessation may reduce the risk of cognitive impairment or dementia. Some studies evaluated the impact of smoking cessation on dementia. A cohort study, including 13,002 community residents in the United States, showed that compared to never smokers, the risk ratio for all-cause dementia was 1.33 (95% CI 1.12, 1.59) for current smokers, 1.24 (95% CI 1.01, 1.52) for those who quit smoking <9 years, and there was no association between quitting smoking for ≥9 years and dementia (15). A longitudinal cohort study in South Korea showed that compared to current smokers, never smokers and ex-smokers who quit for ≥4 years had a 19 and 14% lower risk of developing Alzheimer’s disease, and had a 29 and 32% lower risk of developing vascular dementia within 8 years, respectively (12). Subsequently, Lee et al. (19) conducted a cohort study on atrial fibrillation patients in South Korea. They found that the effect of smoking cessation on lowering the risk of vascular dementia was more pronounced when compared to the risk of Alzheimer’s disease (19). Furthermore, it is suggested that the greater benefits in reducing vascular dementia may be attributed to the rapid reduction in cardiovascular and cerebrovascular risks after quitting smoking (37).

There could be cases where cognitive decline has occurred but is not sufficient for a diagnosis of dementia. Thus, further research on the changes of cognitive score may be more meaningful. However, previous results remain controversial. A cohort study found no significant difference in cognitive function of ex-smokers and current smokers when compared to never smokers (15). A clinical trial in Australia showed that quitting smoking can effectively prevent cognitive decline (16). A study conducted in Germany found that individuals who have quit smoking for a longer duration have higher cognitive scores compared to current smokers (14). It was similar to our conclusion. Due to variations in study population, cognitive assessment tools, research methodologies and other factors, the conclusions derived from previous studies are inconsistent and cannot be broadly generalized. Therefore, we conducted a specific study on Chinese residents, in order to gain further insight into these associations.

Because smoking and cognitive decline are usually chronic, and the lifespan of older adults is already limited, the duration of smoking cessation becomes an important factor to consider. However, there is limited research specifically exploring the relationship of smoking cessation duration and the risk of cognitive impairment. A cohort study in Japan aimed to explore the topic in more detail. The ex-smokers were further divided based on smoking cessation duration: ≤2 years, 3–5 years, 6–10 years, 11–15 years, and >15 years. The results indicated that compared to never smokers, ex-smokers who had quit for ≤2 years still had a higher risk of dementia. Only after quitting smoking for at least 3 years, did the risk of developing dementia become comparable to that of never smokers (13). A study conducted in Germany categorized ex-smokers into four groups based on the duration of smoking cessation: ≤10 years, 11–20 years, 21–30 years, and >30 years. The findings revealed that only ex-smokers who had quit for ≥21 years demonstrated superior cognitive function compared to current smokers (14). We have observed that the selection of grouping nodes when categorizing smoking cessation duration is often subjective, which may affect our assessment of the relationship of specific years of smoking cessation and cognitive function. To minimize this bias, we employed restricted cubic spline analysis and determined the grouping nodes based on the spline trends. Our findings indicated that respondents who had quit smoking for at least 9 years had significantly better cognitive function compared to current smokers.

Our study is the first nationwide research that focuses on the relationship between smoking status, particularly smoking cessation duration, and cognitive function among middle-aged and older residents in Chinese communities. Rigorous quality control measures were implemented at each stage of the CHARLS project (17). The sample is nationally representative and of a large size. We employed a more objective method for grouping than previous studies. To explore the stability and heterogeneity sources of the results, multiple methods were used for analysis. As a widely used cognitive assessment tool, the Mini-Mental State Examination was adopted to facilitate comparisons between studies.

This study does have certain limitations. Firstly, this study utilized cross-sectional data, which limited our opportunity to establish a causal relationship between smoking status and cognitive scores. Secondly, most of the data in this study relied on self-reporting by respondents or informants, which might introduce recall bias and/or reporting biases. To mitigate these potential influences, the CHARLS project team conducted pilot surveys prior to the national survey to validate and revise the questionnaire and survey procedures. Trained interviewers conducted face-to-face data collection. And we excluded respondents with significantly impaired cognitive function to minimize errors stemming from recall bias. Lastly, like all observational epidemiological studies, despite adjusting for all possible confounding factors measured in CHARLS, we could not completely eliminate residual confounding effects because there might be unmeasured and unknown confounding factors.

In conclusion, this study supports the viewpoint that smoking cessation can reduce the risk of cognitive impairment among middle-aged and older residents in Chinese communities. Compared to current smokers, both never smokers and ex-smokers had better cognitive performance. Ex-smokers showed significantly better cognitive function than current smokers only after quitting smoking for at least 9 years. From a trend perspective, respondents with longer smoking cessation duration showed better cognitive performance. This trend tended to level off after 20 years of smoking cessation.

## Data Availability

Publicly available datasets were analyzed in this study. The databases that support the findings of this study are publicly available in CHARLS, and can be downloaded at http://charls.pku.edu.cn/.

## Footnotes

^1^ http://charls.pku.edu.cn/
