# Supplementary material for: Relationship of smoking cessation duration and cognitive function among middle-aged and older adults in China: a national cross-sectional study
Source: Front Public Health. 2025 Jan 7;12:1503152. doi: 10.3389/fpubh.2024.1503152 (PMC11747424; doi:10.3389/fpubh.2024.1503152)
Supplement: Supplementary file 1 [file Table_1.docx]

**Supplementary Table 1. Characteristics of ex-smokers grouped by years from smoking cessation**

|  | ≤2 years  (n=296) | 3-8 years  (n=347) | 9-19 years  (n=287) | ≥20 years  (n=221) | ^*^*P* value |
| --- | --- | --- | --- | --- | --- |
| Age, mean (SD), y | 68.6 (6.4) | 68.4 (6.5) | 68.7 (6.2) | 70.9 (7.6) | <0.001 |
| Male, n (%) | 267 (90.2) | 317 (91.4) | 254 (88.5) | 200 (90.5) | 0.687 |
| Education, n (%) | — | — | — | — | 0.097 |
| Illiterate | 30 (10.1) | 40 (11.5) | 30 (10.5) | 21 (9.5) | — |
| Elementary school | 156 (52.7) | 170 (49.0) | 140 (48.8) | 103 (46.6) | — |
| Middle school | 71 (24.0) | 92 (26.5) | 62 (21.6) | 47 (21.3) | — |
| ≥High school | 39 (13.2) | 45 (13.0) | 55 (19.2) | 50 (22.6) | — |
| Married/living together, n (%) | 262 (88.5) | 307 (88.5) | 245 (85.4) | 184 (83.3) | 0.212 |
| Residence in urban, n (%) | 114 (38.5) | 141 (40.6) | 152 (53.0) | 122 (55.2) | <0.001 |
| Current alcohol intake, n (%) | 111 (37.5) | 136 (39.2) | 112 (39.0) | 73 (34.6) | 0.459 |
| Smoking amount, M(IQR)^†^ | 32.0(34.0) | 36.0(36.0) | 34.0(40.0) | 14.0(22.8) | <0.001 |
| Socially active, n (%) | 133 (44.9) | 79 (22.8) | 62 (21.6) | 54 (24.4) | 0.900 |
| Depressive symptoms, n (%) | 74 (25.0) | 136 (39.2) | 112 (39.0) | 73 (34.6) | 0.115 |
| Chronic pain, n (%) | 82 (27.7) | 65 (18.7) | 62 (21.6) | 38 (17.2) | 0.330 |
| Chronic diseases, mean (SD) | 2.5 (1.7) | 2.4 (1.6) | 2.6 (1.8) | 2.6 (1.9) | 0.496 |
| MMSE score, mean (SD) | 23.0 (5.0) | 23.4 (4.7) | 24.0 (4.1) | 24.2 (4.3) | 0.005 |
| Episodic memory, mean (SD)^‡^ | 7.1 (4.0) | 7.0 (3.8) | 7.5 (3.8) | 7.7 (3.9) | 0.146 |

^*^*P* value, results among the four groups. ^†^Missing n=52, the sample sizes for each group were 293, 331, 272 and 203 respectively. ^‡^Missing n=76, the sample sizes for each group were 268, 330, 271 and 206 respectively.
